# EVER Proteins, Key Elements of the Natural Anti-Human Papillomavirus Barrier, Are Regulated upon T-Cell Activation

**DOI:** 10.1371/journal.pone.0039995

**Published:** 2012-06-28

**Authors:** Maciej Lazarczyk, Cécile Dalard, Myriam Hayder, Loïc Dupre, Béatrice Pignolet, Slawomir Majewski, Francoise Vuillier, Michel Favre, Roland S. Liblau

**Affiliations:** 1 Inserm, U1043, Toulouse, France; 2 CNRS, UMR5282, Toulouse, France; 3 Université de Toulouse, UPS, Centre de Physiopathologie Toulouse Purpan (CPTP), Toulouse, France; 4 Department of Dermatology and Venereology, Medical University of Warsaw, Warsaw, Poland; 5 Institut Pasteur, Unité de Génétique, Papillomavirus et Cancer Humain, Paris, France; Institut de Pharmacologie et de Biologie Structurale, France

## Abstract

Human papillomaviruses (HPV) cause a variety of mucosal and skin lesions ranging from benign proliferations to invasive carcinomas. The clinical manifestations of infection are determined by host-related factors that define the natural anti-HPV barrier. Key elements of this barrier are the EVER1 and EVER2 proteins, as deficiency in either one of the EVER proteins leads to *Epidermodysplasia Verruciformis* (EV), a genodermatosis associated with HPV-induced skin carcinoma. Although EVERs have been shown to regulate zinc homeostasis in keratinocytes, their expression and function in other cell types that may participate to the anti-HPV barrier remain to be investigated. In this work, we demonstrate that *EVER* genes are expressed in different tissues, and most notably in lymphocytes. Interestingly, in contrast to the skin, where *EVER2* transcripts are hardly detectable, *EVER* genes are both abundantly expressed in murine and human T cells. Activation of CD4+ and CD8+ T cells via the TCR triggers a rapid and profound decrease in *EVER* expression, accompanied by an accumulation of free Zn^2+^ ions. Thus, EVER proteins may be involved in the regulation of cellular zinc homeostasis in lymphocytes. Consistent with this hypothesis, we show that the concentration of Zn^2+^ ions is elevated in lymphoblastoid cells or primary T cells from EVER2-deficient patients. Interestingly, we also show that Zn^2+^ excess blocks T-cell activation and proliferation. Therefore, EVER proteins appear as key components of the activation-dependent regulation of Zn^2+^ concentration in T cells. However, the impact of EVER-deficiency in T cells on EV pathogenesis remains to be elucidated.

## Introduction

Papillomaviruses are widespread infectious agents, transmitted by sexual or cutaneous contacts. Human papillomaviruses (HPV) cause a variety of mucosal and skin lesions, ranging from clinically unapparent and benign proliferations such as warts, papillomas or condylomas, to fully invasive cervical or skin carcinomas. A crucial role in determining the clinical outcome of a given HPV infection is played by host-related factors [1], [2]. Notably, the majority of HPV-induced lesions (both skin and mucosal) are spontaneously cleared. In some cases the virus is not eradicated, leading to persistent lesions that may evolve to invasive carcinoma [2]. The nature of this host-virus interplay is largely unknown, but it has been proposed that ill-defined host derived factors could form a natural anti-HPV barrier [3], [4]. This concept is supported by the existence of *Epidermodysplasia Verruciformis* (EV), a rare human genetic disease associated with exquisite sensitivity to papillomaviruses belonging to the beta genus (ß-HPVs) [4]–[6]. EV-suffering patients display a defect in the natural anti-HPV barrier, leading to life-long persistence of skin HPV infections and consequently to the development of diverse epidermal lesions, including skin carcinoma induced by HPV5. The mechanism of this unusual vulnerability to HPV in EV patients and the nature of the deficiency of the anti-HPV barrier remain uncertain. However, we have previously demonstrated that loss-of-function mutations in either of two genes (*EVER1/TMC6* or *EVER2/TMC8*) are responsible for the majority of EV cases [7]–[10]. Recently, we have also shown that EVER1/TMC6 and EVER2/TMC8 proteins are located in the endoplasmic reticulum of keratinocytes, where they form a complex with zinc transporter-1 (ZnT-1), thereby controlling the cellular zinc balance [11].

The concentration of free zinc ions in the cell is extremely small, in the low nanomolar range. As HPV E6 and E7 are zinc-binding proteins, it has been hypothesized that EVER could directly control virus life cycle in keratinocytes by limiting accessibility of free Zn^2+^ ions. Nevertheless, Zn^2+^ ions can influence numerous signal transduction pathways, in different cell types [12]–[14], and zinc imbalance could have, potentially, much broader effects. Based on preliminary transcriptome analyses [15], *EVER* might be ubiquitously expressed, suggesting that its expression in cells other than keratinocytes could contribute to the anti-HPV barrier. Indeed, several clinical observations indirectly suggest that the defect in the natural anti-HPV barrier in EV patients is not limited to epidermis but may potentially involve the adaptive immune system [5]. Notably, EV patients consistently exhibit immune abnormalities, mainly related to defective cell-mediated responses [6], [16]. The responses to HPV antigens and common skin sensitizers (e.g., dinitrochlorobenzene) are compromised in EV patients [16]–[18]. Moreover, EV-like skin eruptions associated with HPV have been only in some instances reported in severely immunocompromised patients, pointing to the involvement of very specific mechanisms in the control of ß-HPVs [19]–[23].

The causes of the immunological defects in EV remain controversial [6], [10]. They could be directly due to the impact of EVER-deficiency in immune cells or secondary to the massive life-long HPV skin infection [24]. Therefore, we decided to assess the expression and function of EVERs in lymphocytes.

## Results and Discussion

**Figure 1:**
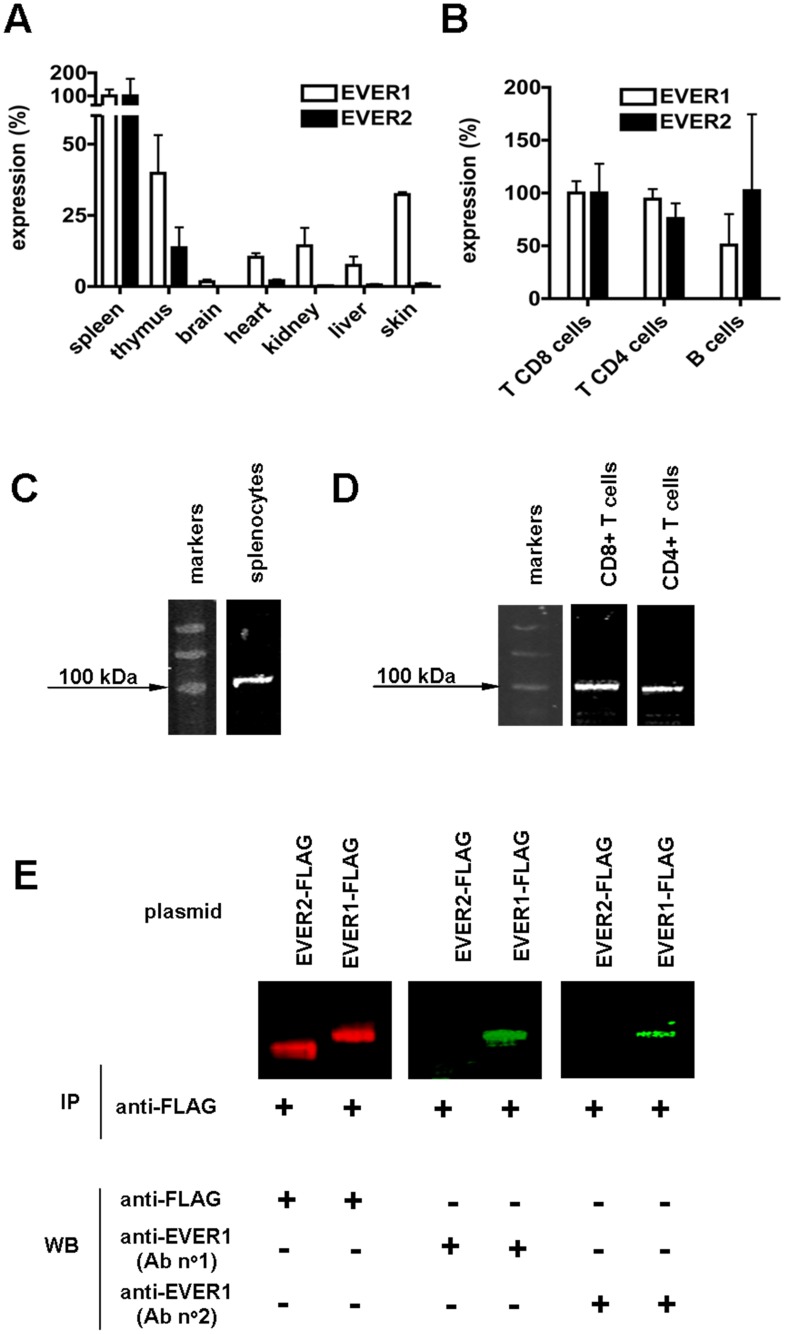
*EVER1* and *EVER2* are expressed in lymphocytes. **A.** Expression of *EVER1* and *EVER2* genes in different mouse organs was assessed by qRT-PCR. Expression in the spleen was set as 100%. For each organ at least 3 independent experiments were performed. **B.** Expression of *EVER1* and *EVER2* genes in murine T and B cells was determined by qRT-PCR. At least 3 independent experiments were performed for each cell type. Expression of EVER1 protein in murine splenocytes (**C**) and purified T cells (**D**) was determined by western blot. An EVER1-specific antibody (Abcam; ab67326) was used. A single band at ≈100 kDa was detected (predicted EVER1 MW = 91 kDa). **E.** 293 T cells were transfected with plasmids encoding either EVER1-FLAG or EVER2-FLAG fusion protein. The fusion protein was immunoprecipitated with anti-FLAG antibody and subsequently a western-blot was performed using the anti-FLAG antibody or two different anti-EVER1 antibodies (Ab n^o^1 - Abcam; ab67326; Ab n°2 - Osenses; OSR00223W).

We first analyzed the expression of *EVER* genes in a panel of freshly collected murine tissues, and in murine and human lymphocyte subsets. Using both classical RT-PCR (data not shown) and quantitative RT-PCR (qRT-PCR), we observed that *EVER1* and *EVER2* were clearly expressed in spleen and thymus (Fig. 1A), as well as in purified murine (Fig. 1B) and human (Fig. S1A, B) lymphocyte populations. *EVER1*, and to a lesser extent *EVER2,* were also expressed in brain, heart, kidney, liver and skin (Fig. 1A). The identity of the amplified bands was confirmed by direct sequencing of the RT-PCR products or by cloning and sequencing of the qRT-PCR products (data not shown). It is noteworthy that the highest expression of *EVER1* and *EVER2* genes was not found in the skin but rather in lymphoid organs (Fig. 1A). The expression of *EVER1* and *EVER2* did not differ significantly between B and T cells (Fig. 1B), or between CD8+ and CD4+ T cells (Fig. 1B, Fig. S1B). Moreover, the amount of *EVER1* and *EVER2* transcripts, as assessed by qRT-PCR, was similar in CD8+ and CD4+ T cell subsets. The mean EVER2/EVER1 expression ratio was 0.96 (n = 4) and 0.77 (n = 6) in CD8+ and CD4+ T cells, respectively. Interestingly, a strikingly different pattern of *EVER* gene expression was observed in the skin where *EVER1* was preferentially expressed whereas *EVER2* was barely detectable (mean EVER2/EVER1 expression ratio 0.07; n = 3).

EVER1- or EVER2-deficiency leads to a clinically identical EV phenotype in humans [7]–[9]. This observation has two important implications. First, it suggests that EVER1 and EVER2 have non-redundant functions. Indeed, the absence of either one of the EVER proteins leads to the dysfunction of the whole EVER complex resulting in zinc imbalance [11]. Second, the cell type(s)/tissue(s) where both genes are expressed, and not those with a selective expression of either EVER1 or EVER2, are most probably essential in EV pathogenesis. In this context, it is tempting to speculate that the lymphoid tissue, which is characterized by a high expression of both *EVER1* and *EVER2* genes, rather than the skin, where the constitutive expression of EVER2 is extremely low (Fig. 1A), could be most relevant to EV pathogenesis.

We next verified by western blot that EVER proteins were expressed in spleen and freshly purified CD4+ or CD8+ T cells. A single band of approximately 100 kDa was detected using a commercially available antibody against murine EVER1 (Fig. 1C, D). To ensure that the antibody used was specific, and that it could discriminate between the two related EVER1 and EVER2 proteins, we employed two complementary strategies. First, the expression of EVER1 was confirmed with a second antibody recognizing a distinct epitope of EVER1 (Osenses; OSR00223W). A single band of approximately 100 kDa was detected with this antibody in spleen and in CD8+ T cell lysates (data not shown). Second, we tested whether these two anti-EVER1 antibodies specifically recognized EVER1. To this end, 293 T cells were transfected with plasmids encoding either EVER1-FLAG or EVER2-FLAG fusion proteins. The fusion proteins were subsequently precipitated using the anti-FLAG antibody and western blot analyses were performed. We were able to detect both EVER1-FLAG and EVER2-FLAG fusion proteins with the anti-FLAG antibody. However, the two anti-EVER1 antibodies recognized a single band (corresponding to MW ≈ 85 kDa) in cells expressing EVER1-FLAG, but not in cells expressing EVER2-FLAG (Fig. 1E), demonstrating their specificity. We attempted to verify by the same approach the expression of the EVER2 protein in T cells but the anti-EVER2 antibody tested (Abcam, ab70002) did not work in our hands (data not shown).

**Figure 2:**
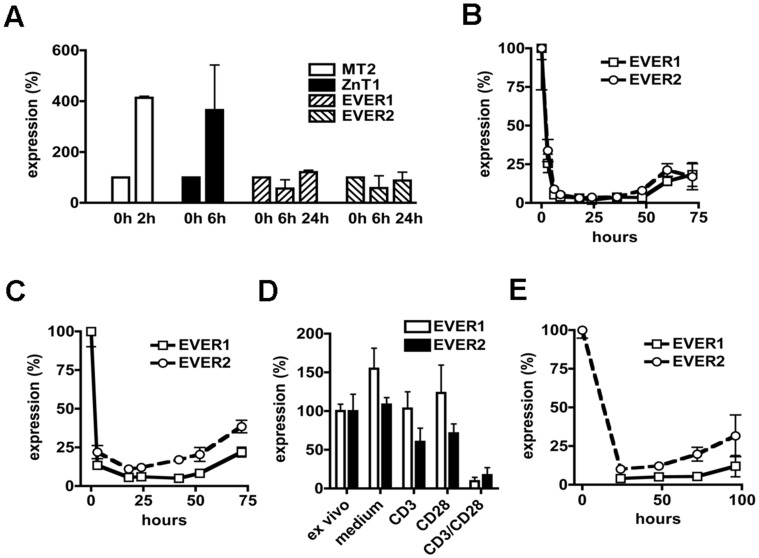
*EVER1* and *EVER2* expression is strongly down-regulated in T cells following TCR-mediated activation. **A.** Purified murine naive CD8+ T cells were incubated in 100 µM zinc for the indicated period of time. Thereafter the expression of metallothionein 2 (*MT-2*), zinc transporter 1 (*ZnT-1*) as well as *EVER1* and *EVER2* was assessed by qRT-PCR. Data are from 3 independent experiments. Purified murine naive CD8+ T cells (**B**) or CD4+ T cells (**C**) were activated with immobilized anti-CD3 and anti-CD28 Abs, in the presence of IL-2 (1 ng/ml) and IL-12 (10 ng/ml). Expression of *EVER* genes in the course of the activation was determined by qRT-PCR at the indicated time points. Data are representative of 3 independent experiments. **D.** Purified murine naive CD8+ T cells were incubated in medium or activated by anti-CD3 (CD3), anti-CD28 (CD28) or by a combination of anti-CD3 and anti-CD28 (CD3/CD28) Abs. After 24 h, the expression of *EVER1* and *EVER2* was determined by qRT-PCR. Freshly purified naive CD8+ T cells (ex vivo) were used as the reference 100% value. Data are from 3 independent experiments. **E.** Purified naive TCR-transgenic CD8+ T cells were activated by their cognate HA_512–520_ peptide in the presence of irradiated T cell-depleted syngeneic splenocytes as antigen-presenting cells. At the indicated time points, CD8+ T cells recovered by MACS purification, and expression of *EVER1* and *EVER2* in these cells was determined by qRT-PCR. Data are representative of 3 independent experiments.

Since EVER proteins form a complex with ZnT-1 [4], [11], a well-characterized zinc transporter whose expression is enhanced by Zn^2+^ ions through the MTF-1 transcription factor [25], [26], we evaluated whether transcription of *EVER1* and *EVER2* in T cells could also be regulated by zinc. Whereas expression of two zinc-inducible genes (*MT-2* and *ZnT-1*) was clearly increased 2 or 6 hours after exposition to a non-toxic concentration of zinc (100 µM), expression of *EVER* genes remained stable following 6- and even 24-hour exposition to zinc (Fig. 2A).

We next tested the impact of *in vitro* T-cell activation and differentiation on EVER expression. *EVER1* and *EVER2* were constitutively expressed in purified naive CD8+ (Fig. 2B) and CD4+ (Fig. 2C) T cells. Interestingly, activation of these T cells using anti-CD3 and anti-CD28 antibodies led to a rapid and profound decrease in *EVER1* and *EVER2* mRNA levels. The drop in *EVER1* and *EVER2* expression was detected as early as 3 hours after activation (Fig. 2B, C). This decreased expression of *EVER* genes was maintained for at least 3 days (Fig. 2B, C). A similar *EVER1* and *EVER2* mRNA down-regulation was found in human T cells following TCR/CD28–dependent activation (Fig. S2A, B). The TCR and CD28 exerted a synergistic effect on the down-modulation of *EVER* genes, as triggering of either TCR or CD28 alone had little or no effect (Fig. 2D). We then validated these data in an antigen-driven T-cell activation system, using naive CD8+ T cells specific for an *influenza* virus hemagglutinin peptide (HA_512–520_; IYSTVASSL), originating from CL4-TCR transgenic mice [27], [28]. Activation of purified TCR-transgenic CD8+ T cells with syngeneic antigen-presenting cells loaded with the cognate HA_512–520_ peptide was also associated with a clear and long-lasting decrease in the expression of both *EVER* genes (Fig. 2E).

The exact mechanism of the down-regulation of *EVER* expression in response to T-cell activation remains unknown. Interestingly, the reduction in the amount of *EVER* transcripts is rapid, suggesting that *EVER* mRNA might be relatively unstable. Probably, due to its rapid turnover, the suppression of the transcription of the corresponding gene would result in such a rapid reduction in the mRNA quantity. Regardless of the exact molecular basis of this observation, the role of EVER1 and EVER2 in T cells and the functional significance of the modulation of their expression constitute an important unanswered question.

**Figure 3:**
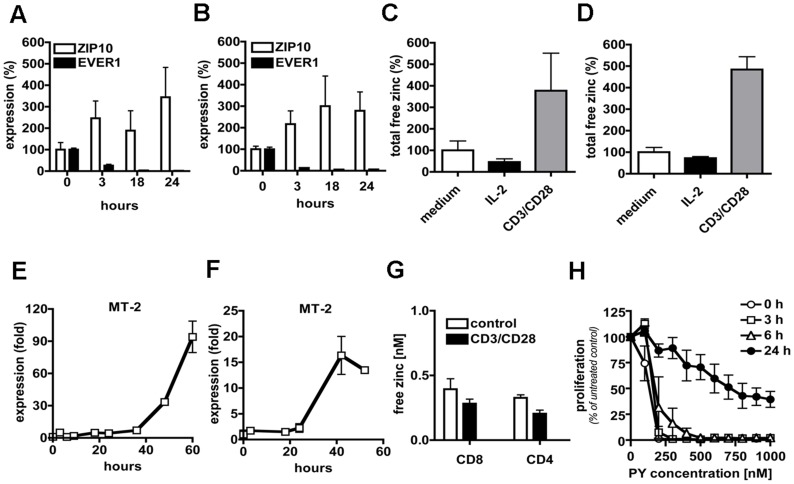
Zinc homeostasis in T cells. Purified murine naive CD8+ (A) or CD4+ T cells (B) were activated with immobilized anti-CD3 and anti-CD28 Abs and expression of the *ZIP10* zinc transporter and *EVER1* genes was determined by qRT-PCR at the indicated time points. Data are from 3 independent experiments. Total amount of free zinc was determined in murine CD8+ (**C**) or CD4+ T cells (**D**) after 24 h incubation in standard medium (medium), in medium supplemented with IL-2 (IL-2), or with anti-CD3/anti-CD28 Abs in the presence of IL-2 (CD3/CD28). The content of free zinc was measured by flow cytometry with FluoZin-3, a zinc ion-specific indicator. The total amount of free zinc per cell was calculated as described in materials and methods and normalized for the unstimulated cells (medium) values (100%). Purified naive CD8+ (**E**) or CD4+ T cells (**F**) were stimulated by immobilized anti-CD3 and anti-CD28 Abs in the presence of IL-2 and IL-12. The expression of *metallothionein 2* (MT-2) was assessed by qRT-PCR. Data are representative of 3 independent experiments. **G**. Murine naive CD8+ or CD4+ T cells were activated (CD3/CD28) or not (control) by immobilized anti-CD3 and anti-CD28 Abs for 24 h The concentration of free zinc was then measured by flow cytometry with FluoZin-3. **H**. Purified murine naive CD8+ T cells were activated with immobilized anti-CD3 and anti-CD28 Abs. At the indicated time the zinc ionophore pyrithione (PY) was added to the medium at different concentrations. Radiolabeled thymidine was added 24 hrs after initiation of the cell culture, and proliferation was assessed on the basis of the thymidine incorporation 24 h later.

Cellular zinc homeostasis is tightly controlled by an elaborate system composed of different zinc sensors (like MTF-1), transporters (ZnT and ZIP families), and buffering proteins such as metallothioneins [29]. It has been suggested that ZnT-1 and the EVER/ZnT-1 complex, like the other members of the ZnT family, could be involved in the transport of Zn^2+^ ions out of the cytoplasm, in order to maintain the low Zn^2+^ cytoplasmic level in spite of the large gradient of this metal across the plasma membrane [4], [11], [29]. Based on our data, we propose that the EVER complex is involved in the regulation of zinc balance in T cells, and that down-modulation of the *EVER* expression during T-cell activation could impact zinc homeostasis. In the course of CD8+ and CD4+ T-cell activation, we observed a decreased expression of *EVER1* and *EVER*2 as well as of *ZnT-1* (data not shown), with concomitant up-regulation of *ZIP10* (Fig. 3A, B) and *ZIP6* (Fig. S3A, B) expression. ZIP10 and ZIP6 are plasma membrane transporters responsible for Zn^2+^ influx into the cell, and in particular in T cells [30], [31]. This coordinated reorganization of zinc transporter expression is compatible with a program of zinc accumulation in the cytosol of activated T cells. Indeed, we confirmed that the total amount of free Zn^2+^ ions per cell was significantly increased 24 h after TCR- and CD28-dependent activation (Fig. 3C, D), and maintained at this level for at least 72 h (data not shown). The mechanism and functional significance of such an accumulation remain uncertain given the very limited knowledge on the physiological role of free Zn^2+^ ions in T cells. However, this zinc accumulation could be related to the massive changes in lymphocyte volume (about 10 times, based on the changes of the forward side scatter in flow cytometry) and metabolic activity during blast formation. Thus, at least two non-mutually exclusive hypotheses could be formulated. First, zinc accumulation could be a response to the increased needs of this metal for the newly synthesized zinc-binding proteins. Second, it might constitute a compensatory mechanism in order to maintain a constant free Zn^2+^ ion concentration during the massive enlargement of the stimulated T cells.

**Figure 4:**
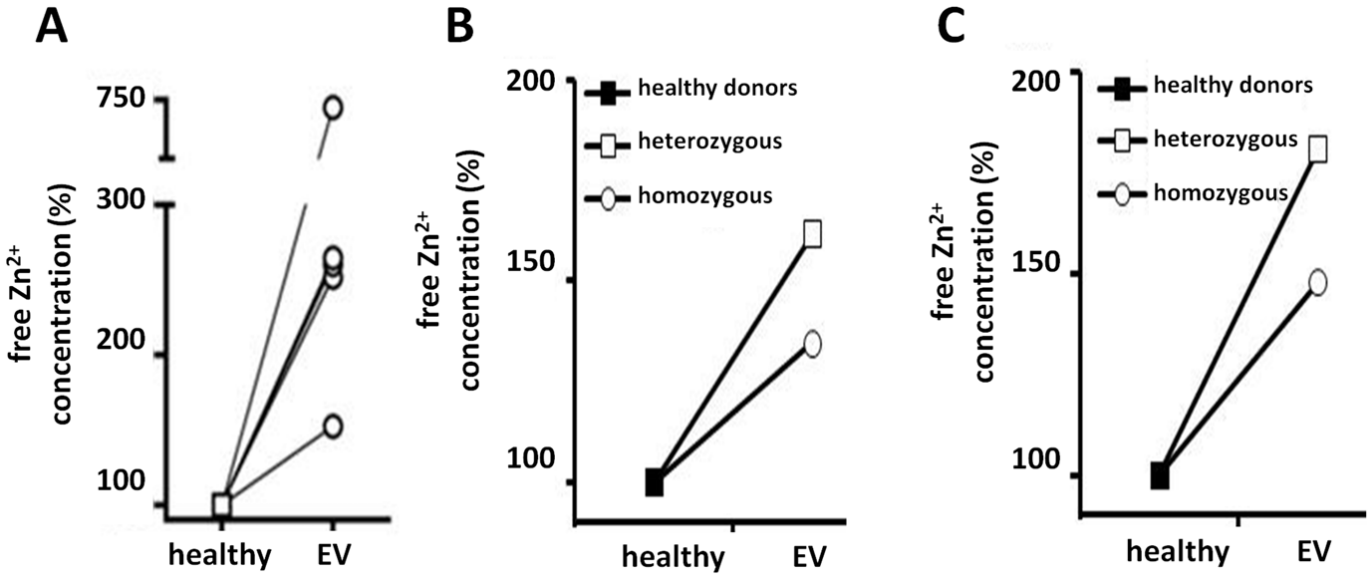
Increased cellular zinc concentration in lymphocytes from EVER2-deficient patients. **A.** The concentration of free zinc was measured with FluoZin-3 in two EBV-transformed lymphoblastoid cell lines, derived from two individuals from the same family. The healthy subject carried wild-type copies of *EVER1* and *EVER2* genes (healthy), and the patient suffering from *Epidermodysplasia Verruciformis* had a homozygous nonsense mutation (del275A) in the *EVER1* gene (EV). For each line, 5 independent experiments were performed and the relative values obtained in each of these experiments were plotted against the mean concentration of free Zn^2+^ in the control cells, taken as 100%. A bilateral paired Student’s t test analysis indicates a p value of 0.099. **B–C.** Primary T cell lines were generated from two patients, one with full-blown EV and homozygous for the *EVER2* T150fdX3 mutation and one with transient EV-like clinical presentation and heterozygous for the *EVER2* T150fdX3 mutation, as well as from two unrelated healthy individuals carrying wild-type copies of *EVER1* and *EVER2* genes (healthy). The concentration of free zinc was measured with FluoZin-3 in CD4+ T cells maintained in standard culture medium (**B**) or incubated for 18 h in medium supplemented with 100 µM of zinc (**C**). For each line, 2 independent experiments were performed and the mean values for each EVER2-deficient line were plotted against the mean concentration of free Zn^2+^ in the two control lines (healthy) (100%).

Recent findings, showing that Zn^2+^ ions play an important role in signal transduction and can even serve as a classical second messenger [32]–[34], highlight the need for tight control of the intracellular free zinc pool. Indeed, we observed that the aforementioned modulation in zinc transporter expression in activated CD4+ and CD8+ T cells is accompanied by massive transcription of *MT-2* (Fig. 3E, F), encoding for the major zinc-buffering metallothionein [35], [36]. Finally, despite the increase in the total zinc content per cell following TCR/CD28 triggering (Fig. 3C, D), the intracellular concentration of free zinc remained remarkably stable (Fig. 3G). In this context, it is tempting to speculate that the lack of either EVER proteins could disrupt the EVER/ZnT-1 complex and consequently impose zinc imbalance in T cells as previously demonstrated in keratinocytes [11]. To assess the impact of the EVER-deficiency on zinc homeostasis in lymphocytes, we first measured the concentrations of free Zn^2+^ in EBV-transformed B cells from an EV patient (*EVER1* Del275A) and from a healthy relative using FluoZin-3, a zinc ion-specific fluorescent probe, and a flow cytometric assay. In five independent measurements, the zinc concentration was higher in EVER-deficient than in control cell lines (Fig. 4A). These results suggest that EVERs could be involved in the regulation of cellular zinc homeostasis.

To then address the contribution of EVER proteins for zinc balance in primary lymphocytes, untransformed T cell lines were generated from PBMC of two healthy individuals and two patients, one with full-blown EV with homozygous *EVER2* T150fdX3 mutation and one heterozygous for the *EVER2* T150fdX3 mutation. From a clinical point of view, this heterozygous patient clearly presented transiently EV-like eruptions and was HPV3 and HPV5 positive in the lesions (see Materials and Methods). Interestingly, high HPV5 viral load was recently described in non-symptomatic family members of an EV patient with heterozygous EVER2 13-nucleotide deletion [37]. These data suggest that heterozygous EVER2 mutation, and the resulting decrease in protein expression, favors HPV5 replication. This may explain the transient EV-like clinical presentation of this heterozygous patient. Using the FluoZin-3 based cytometric assay, two independent measurements of free zinc in CD4+ T cells were performed, side-by-side for all the lines. The mean free Zn^2+^ concentration in EV-derived CD4+ T cells was higher than in CD4+ T cells from healthy donors (Fig. 4B). This higher concentration of free zinc in EVER-deficient CD4+ T cells was maintained following overnight incubation of the cells with a nontoxic zinc concentration (100 µM) (Fig. 4C). Although derived from a small number of patients, these data suggest that EVER proteins might be involved in the control of zinc homeostasis in lymphocytes. At least two different types of intracellular zinc signaling occur in T cells. One happens extremely rapidly after TCR triggering as a result of zinc transporter activity. The other depends on transcriptional changes in expression of zinc importers and exporters that increase the cellular zinc content in a delayed and durable manner. In this study, we have monitored the second type of zinc signal but it would be most interesting to assess whether there is any impact of EVER-deficiency on the early and focal T cell zinc fluxes upon TCR activation. Further studies on a larger cohort of EV patients and/or on *EVER* knock-out mice should help settle this issue.

Since the concentration of free Zn^2+^ is tightly controlled during lymphocyte activation (Fig. 3G) and EVER-deficiency might increase the concentration of unbound zinc ions (Fig. 4), one burning question relates to the biological consequence of the zinc imbalance in T cells. During T-cell activation zinc acts at different levels. Zn^2+^ participates in the interaction between Lck and CD4 and CD8 co-receptors, stabilizes Lck dimerization, and is likely involved in the activation of protein kinase C [38]–[40]. It has been shown that Zn^2+^ is released from lysosomes upon T-cell activation [32] and that it participates to IL-2R-mediated signaling through the regulation of ERK activity [32]. Moreover, zinc influx from extracellular sources is induced within minutes of TCR triggering in primary T cells, with compartmentalized high zinc concentration under the zone of the T cell/APC contact. This local increase in cytoplasmic zinc influences proximal TCR signaling resulting in enhanced proliferative responses to suboptimal stimuli [31]. On the other hand, Tanaka and colleagues have reported that excess of zinc suppresses mitogen-activated IL-2 production in lymphocytes [41]. Collectively, the current data indicate that Zn^2+^ ions play a clear role in signal transduction and T-cell activation, and any deviation in free zinc concentration is likely to impair these processes [42]. This, in turn, highlights the importance of the system that protects the cells from the high concentration of Zn^2+^ in the extra-cellular milieu. Interestingly, when we disrupted this zinc homeostasis by incubating T cells with non-toxic concentrations of a zinc ionophore (pyrithione), the CD3/CD28-dependent proliferation was inhibited, as demonstrated by thymidine incorporation (Fig. 3H) and CFSE dilution assay (data not shown). Naive T cells appear more sensitive to zinc than activated T cells (Fig. 3H), even though this inhibition is only temporary lasting cells enter into mitosis 24 hours after exposure to pyrithione (data not shown). The reason of the higher sensitivity to zinc of naive T cells remains to be elucidated. It is likely that activated T cells are more resistant to excess of zinc, since they have already reorganized the system controlling zinc homeostasis, and notably exhibit increased expression of zinc buffering proteins (metallothioneins). Taking into account the known properties of ZnT-1 as a zinc transporter, and our observations of increased concentration of Zn^2+^ in EVER-deficient lymphocytes (Fig. 4), it seems probable that the ZnT-1/EVER complex participates in the maintenance of low levels of free zinc in lymphocytes. It is tempting to speculate that EVER-deficiency, by shifting zinc homeostasis, could have an impact on T-cell activation, especially in naive T cells, in which EVER expression is the highest. Certainly, the clinical manifestations of EVER-deficiency suggest that such an impact does not interfere with crucial steps of T-cell activation, as EV patients respond normally to pathogens other than HPV. Indeed, we did not observe any striking differences in the rate of culture expansion during preparation of the primary T cell lines from EV patients and healthy controls (data not shown).

In conclusion, we have demonstrated that *EVER* genes are expressed at high levels in T and B cells. Their expression in T cells is sharply decreased upon TCR/CD28 stimulation, as a part of the global activation-dependent reorganization of the molecular machinery controlling cellular zinc homeostasis. Indeed, the elevated intracellular amount of free Zn^2+^ ions is associated with decreased expression of *ZnT-1* and increased expression of *Zip10* and *Zip6* in activated T cells. The preliminary data from EV patient-derived cell lines suggest that EVER might be involved in maintaining a constant low level of free zinc in lymphocytes. Altogether, these results support our previously formulated hypothesis [4] that the natural anti-HPV barrier, of which EVER proteins constitute an essential part, might comprise at least two arms: keratinocytes and lymphocytes. Although still speculative, we suggest that EVER-deficiency in T cells may contribute to susceptibility to HPV infection, possibly in combination with EVER-deficiency in keratinocytes. However, the exact mechanism of the impact of EVER-deficiency on T cell function as well as the reason for the extreme selectivity of this barrier towards HPV remains to be elucidated.

## Materials and Methods

### Mice

For all experiments 8 to 12 week-old female BALB/c mice were used. For antigen-driven T-cell activation, we used the CL4-TCR transgenic BALB/c mice, which express a TCR specific for the influenza virus HA_512–520_ peptide in the context of H2-K^d^ on most CD8+ T cells [27], [28]. All breeding and experimental procedures were carried out in accordance with European Union guidelines and were approved by the regional ethics committee N°MP/18/26/04/04.

### T Cell Purification and Culture

For T cell cultures, spleens and lymph nodes were collected and naive CD4+ or CD8+ T cells (CD4^+^ CD25^−^ CD62L^+^ and CD8^+^ CD25^−^ CD62L^+^, respectively) were purified by MACS (Milteny Biotec) according to the manufacturer’s protocol (≥92% or 94% purity, respectively). The purified naive T cells were activated by immobilized anti-CD3 (BD Biosciences, clone 145-2C11), anti-CD28 (BD Biosciences, clone 37.51) antibodies individually, or combination CD3/CD28 beads were used (Dynabeads mouse CD3/CD28 T cell expander, Invitrogen). Antigen-specific stimulation was performed with CL4-TCR T cells co-cultured with irradiated, T cell-depleted, syngeneic splenocytes loaded with the cognate peptide. For the subsequent PCR analysis transgenic T cells were isolated by MACS (Milteny Biotec) using biotinylated Thy1.2 mAb (BD Pharmingen, clone 53-2.1) combined with streptavidin microbeads (Milteny Biotec).

For human T cell cultures, PBMC were obtained from buffy coat preparations from anonymous healthy donors, from the Purpan university hospital blood bank (Toulouse, France). Naive CD8+ T cells were sorted by MACS from PBMC with naive CD8+ T cell isolation kit (Milteny Biotec). Human CD4+ T cells (TCR+,CD4+,CD45RC^High^ and TCR+,CD4+,CD45RC^low^) were purified by FACS. The cells were thereafter stimulated by anti-CD3 and anti-CD28 beads (Dynabeads human T activator CD3/CD28, Invitrogen).

### EBV-transformed Human Lymphocytes

The lines have previously been generated [9] using PBMC from an EV suffering patient (*EVER2* Del275A, B. Bouadjar, P. Cassonnet, M. Favre, G. Orth, M. Ramoz, unpublished results) or from healthy relatives (homozygous *EVER2* wt) by transformation with EBV. The cells were cultured in standard RPMI (Invitrogen) medium supplemented with 10% FBS (Invitrogen). The *EVER2* genotype of each of the lines was confirmed by sequencing directly before the experiments.

### Primary Human T Cell Lines

One patient presented with full-blown EV and was homozygous for the *EVER2* T150fdX3 mutation (P. Cassonnet, M. Favre, S. Jablonska, S. Majewski, G. Orth, M. Ramoz, unpublished results). Her daughter was heterozygous for the *EVER2* T150fdX3 mutation and had developed transient EV-like symptoms. Her first skin lesions were plane warts on the dorsum of the hands that appeared at the age of 7 yrs. The lesions were treated successfully by liquid nitrogen freezing. However at the age of 14 years new extensive skin lesions developed on the dorsum of the hands and feet, as well as on the trunk. These lesions were much more abundant, some of them larger than typical plane warts. Some of the lesions were confluent with uneven polycystic outlines. The lesions persisted for about 2 years and disappeared after several courses of cryotherapy. In the skin lesions DNA of HPV3 and HPV5 was detected. Polyclonal T cell lines were generated from PBMC of the 2 patients or healthy controls, as previously described [43]. Briefly, mixed lymphocyte reaction cultures of PBMC (5×10^5^ cells/ml) and irradiated allogenic PBMC from 2 different donors (10^6^ cells/ml) were performed. The lines were cultured in IMDM (Cambrex Bio Science) supplemented with 1 µg/ml PHA, and 100 IU/ml recombinant human IL-2 (Chiron), 10% YSSEL medium (Dyaclone), 5% FBS (Cambrex Bio science), and penicillin/streptomycin (Bristol-Myers Squibb). T cells were restimulated as described every 2 weeks. T cells were used in this study after one stimulation cycle. The study was approved by the Ethics Committee at the Medical University of Warsaw. Written informed consent was received from all participants.

### RT-PCR

RNA from the cell cultures or organs was isolated by RNeasy mini kit (Quiagen), and reverse transcription (SuperScript III first-strand synthesis system for RT-PCR, Invitrogen) was performed, according to the manufacturer’s instructions. The obtained cDNA was used as a template in classical PCR (RT-PCR; Taq Polymerase, Q-BIOgene) or in quantitative PCR (qRT-PCR; qPCR mastermix plus for SYBRgreen, Eurogentec). The primers used for the assessment of the *EVER* genes expression were designed using primer3 software (http://frodo.wi.mit.edu/primer3/) and their validity was confirmed by direct sequencing of the PCR product (RT-PCR) or by the sequencing of the qRT-PCR products cloned into the standard vector (Topo TA cloning kit for sequencing, Invitrogen). The primers used for qRT-PCR on mouse samples were Ever1_F : 5′CTCAGCTGAAGGAGCTGTTG3’, Ever1_R : 5′CTCTGAGAAGGTGAGGACAGC3′; Ever2_F : 5′GCTTCATCCGGGAACTGC3′, Ever2_R : 5′AGAGGCCCCCAATCTCGTA3′; Znt1_F : 5′GGCCAACACCAGCAATTC3′, Znt1_R : 5′CTTCCGCTTCCAGATTGTCA3′; MT-2_F : 5′AACTCTTCAAACCGATCTCTCGT3′, MT-2_R : 5′AAGTACATTTGCATTGTTTGCATT3′; Zip6_F: 5′GATGGTGATCATGGGCGAC3′, Zip6_R: 5′CTGTTTTGCTAAAGGCTGG3′; Zip10_F : 5′GAGCGGAGAGGAGATGCAC3′, Zip10_R : 5′TCCCACGATGATGTCTGTGA3′; HPRT_F : 5′TGACACTGGTAAAACAATGCAAACT3′, HPRT_R : 5′AACAAAGTCTGGCCTGTATCCAA3′. The primers used for qRT-PCR on human cells were Ever1_F : 5′GTGGCCTGCCCTACAACAT3′, Ever1_R : 5′CCGAAAGAGTGAGCCATGC3′; Ever2_F : 5′CAAGAAGTACACCCTCCTGAAGA3′, Ever2_B : 5′GAGGAGTGGATGCTGCTGAC3′; GAPDH_F : 5′ACGGATTTGGTCGTATTGGGC3′, GAPDH_B : 5′TTGACGGTGCCATGGAATTTG3′.

### Transfections

293 T cells were cultured in DMEM medium (Invitrogen) supplemented with 10% FBS. They were transfected with plasmid encoding EVER1-FLAG or EVER2- FLAG fusion protein using FuGENE-6 (Roche), according to the manufacturer’s instructions. The cells were cultured for 24 h before analysis of EVER protein expression.

### Western Blot Analysis

Primary T cells or transfected 293 cells were lysed using RIPA buffer (10 mM Tris HCL ph 7,5, 50 mM NaCl, 1% Triton X100, 30 mM Na_4_P_2_O_7_, 5 µM ZnCl_2_, 10% Glycerol, 0,1% SDS, 50 mM NaF, 1 nM Na_3_VO_4_, 1 mM DTT, and Roche mini complete protease inhibitor). The crude lysates (or precipitated proteins, depending on the experiment) were migrated on 10% polyacrylamide gels (Novex, Invitrogen) and transferred onto nitrocellulose membranes (Amersham Biosciences). The following primary antibodies were used: polyclonal Ab anti-EVER1 (Osenses ref: OSR00223W), polyclonal Ab anti-EVER1 (Abcam ref: ab67326), anti-ß-actin (Sigma, clone AC-15) and anti-FLAG (Sigma, clone M2). The following secondary antibodies were used anti-rabbit (Li-Cor Biosciences ref 926-32211) and anti-mouse (Li-Cor Biosciences ref: 926-32220) labeled with DyLight fluor dyes (IR DYE 800 and IR DYE 680, respectively) and the signal was detected using the LI-COR Odyssey Infrared Imaging System (LI-COR Biosciences).

For the precipitation of EVER1-FLAG and EVER2-FLAG proteins, we employed the standard immunoprecipitation method with the anti-FLAG antibody and Protein G PLUS agarose (Santa Cruz Biotechnology, ref: sc2002).

### Free Zinc Concentration Measurements

Free zinc concentration in lymphocytes was measured by flow cytometry as previously described [44]. Briefly, the cells were loaded for 30 min. with FluoZin-3 (Invitrogen, ref: F-24195), a zinc ion-specific fluorescent probe. Each experiment contained 3 samples: the “minimum” control, the “maximum” control and the experimental sample. The “minimum” control cells were subsequently incubated with zinc chelator (TPEN; 100 µM - Sigma – ref: 87641). The “maximum” control cells with zinc ionophore (pyrithione; 50 µM - Sigma– ref: 63844) and 100 µM ZnSO4 (Sigma, ref: Z-0251). The zinc concentration was calculated on the basis of the median fluorescence intensity, according to a previously published formula [43]. The changes in the total cellular free zinc content during T-cell activation was assessed by taking into account the changes in the zinc concentration and the modifications of the mean cell volume (estimated according to the formula: VΔ = (FSC_1_)^3^÷(FSC_2_)^3^×100%, where VΔ is a mean volume change in the time and FSC_1_ and FSC_2_ are forward side scatter values measure at two different time points).

### H^3^-thymidine Incorporation Assay

Purified murine naive CD8+ T cells were seeded in 96-well plates and stimulated using anti-CD3 and anti-CD28 beads (Dynabeads mouse CD3/CD28 T cell expander, Invitrogen). At the start of the culture or after 3, 6 or 24 h of stimulation, the zinc ionophore (pyrithione, Sigma, ref: 63844) was added at the indicated concentrations. After 24 h, H^3^-thymidine was added followed by 18 h incubation. Cell associated radioactivity was measured with a Beta counter (PerkinElmer, 1450 LSC & Luminescence counter, MicroBeta Trilux).

## Supporting Information

Figure S1 **Expression of** ***EVER1*** **and** ***EVER2*** **in human T cells.** Expression of *EVER1* and *EVER2* genes was assessed in freshly MACS-purified human CD4+ or CD8+ T cells by **(A)** conventional RT-PCR (2 independent experiments) or **(B)** qRT-PCR (3 independent experiments).
(TIF)

Figure S2 ***EVER1*** **and** ***EVER2*** **expression is strongly down-regulated in human T cells following TCR-mediated activation.** Freshly purified human naive CD8+ **(A)** or CD4+ T cells **(B)** were activated with immobilized anti-CD3 and anti-CD28 Abs. Expression of *EVER* genes was determined by qRT-PCR at the indicated time points in the course of activation. Data are from 3 independent experiments.
(TIF)

Figure S3 **ZIP6 zinc transporter expression is up-regulated in T cells following TCR-mediated activation.** Purified murine naive CD8+ (**A**) or CD4+ T cells (**B**) were activated with immobilized anti-CD3 and anti-CD28 Abs and expression of the *ZIP6* zinc transporter, *EVER1* and *EVER2* genes was determined by qRT-PCR at the indicated time points. Data are from 3 independent experiments.
(TIF)
